# Supplementary material for: Content-rich biological network constructed by mining PubMed abstracts
Source: BMC Bioinformatics. 2004 Oct 8;5:147. doi: 10.1186/1471-2105-5-147 (PMC528731; doi:10.1186/1471-2105-5-147)
Supplement: Additional File 2 — The original results of the above study (non-essential files are deleted to keep the file size under the limit set by BMC bioinformatics). [file 1471-2105-5-147-S2.bz2 › chilibotAdditionalFile2/dip20/249ID7760852E1003/html/RARA_RXRA.html]

 


 **RARA** and **RXRA** 
  
Found 92 abstracts in PubMed, retrieved 20.  
 

 What does Google say? 
 PDF only 
| .edu only 

---

**Interactive relationship** (e.g. stimulation, inhibition, etc)

**Neutral relationship**- The recent discovery of the interaction between nuclear IGFBP 3 and 9 cis retinoic acid receptor alpha  [ **RARA** ]  retinoid X receptor alpha  [ **RXRA** ]  RXRalpha, a nuclear receptor, and its involvement in the regulation of transcriptional signaling and apoptosis represents an important paradigm shift in the understanding of IGFBP function.  Ref: 12379488 J Endocrinol, 2002

**Non-interactive relationship** (e.g. studied together, co-existance, homology, etc.)

- Increased retinoic acid responsiveness in lung carcinoma cells that are nonresponsive despite the presence of endogenous retinoic acid receptor RAR  [ **RARA** ]  beta by expression of exogenous retinoid receptors retinoid X receptor alpha  [ **RXRA** ] , RAR alpha  [ **RARA** ] , and RAR  [ **RARA** ]  gamma.  Ref: 11212249 Cancer Res, 2001
- Moreover, DMS induced the expression of retinoic acid receptor alpha  [ **RARA** ]  and retinoid X receptor alpha  [ **RXRA** ] .  Ref: 11520048 Biochem Biophys Res Commun, 2001
- The two nuclear receptor binding motifs within the PBREM effectively bound PXR as a heterodimer with the 9 cis retinoic acid receptor alpha  [ **RARA** ]  NR2B1  [ **RXRA** ] .  Ref: 11502872 Mol Pharmacol, 2001
- Reverse transcription polymerase chain reaction analysis showed that the retinoic acid receptors RAR alpha  [ **RARA** ] , RAR  [ **RARA** ]  gamma, and retinoid X receptor alpha  [ **RXRA** ]  were present in the cells.  Ref: 11678760 BJU Int, 2001
- Furthermore, prolongation of NR1a NR2A responses was similar to that of responses from NR1b  [ **RARA** ]  NR2B  [ **RXRA** ]  receptor, a subtype characterized by reduced desensitization.  Ref: 11249958 Neuropharmacology, 2001
- Whole cell voltage clamp recordings were performed to assess the ability of conantokin G, conantokin T, and a 17 residue truncated form of conantokin R to inhibit N methyl d aspartate evoked currents in human embryonic kidney 293 cells transiently expressing various combinations of NR1a, NR1b  [ **RARA** ] , NR2A, and NR2B  [ **RXRA** ]  receptor subunits.  Ref: 11335724 J Biol Chem, 2001
- PP1c specifically interacts with VDR but not retinoic acid receptor alpha  [ **RARA** ]  and retinoid X receptor alpha  [ **RXRA** ]  in yeast.  Ref: 12036952 J Biol Chem, 2002
- CONCLUSIONS Bexarotene treatment at clinically relevant concentrations causes apoptosis of CTCL cell lines in association with activation of caspase 3 and cleavage of poly ADP Ribose polymerase, as well as down regulation of retinoid X receptor alpha  [ **RXRA** ] , retinoic acid receptor alpha  [ **RARA** ] , and survivin.  Ref: 12006543 Clin Cancer Res, 2002
- Bexarotene treatment suppressed the expression of retinoid X receptor alpha  [ **RXRA** ]  and retinoic acid receptor alpha  [ **RARA** ]  proteins in all three lines compared with untreated controls.  Ref: 12006543 Clin Cancer Res, 2002
- Sequence comparisons and phylogenetic analyses reveal that these ascidian nuclear receptors are representative of five of the six previously defined nuclear receptor subfamilies and are apparent homologues of retinoic acid NR1B  [ **RARA** ] , retinoid X NR2B  [ **RXRA** ] , peroxisome proliferator activated NR1C, estrogen related NR3B, neuron derived orphan NOR NR4A3, nuclear orphan NR4A, TR2 orphan NR2C1 and COUP orphan NR2F3 receptors.  Ref: 12141458 Int J Dev Biol, 2002
- These results, differential proteolysis sensitivity assays, and glutathione S transferase pulldown experiments demonstrate that, unlike AHPN or the natural or standard synthetic retinoids, MM11453 does not behave as a RAR  [ **RARA** ]  or retinoid X receptor alpha  [ **RXRA** ]  transcriptional agonist.  Ref: 11406543 Cancer Res, 2001
